# Vitamin B Complex Encapsulation in Bacterial Nanocellulose: A Novel System for Heat and Chemical Stabilization in Food Products

**DOI:** 10.3390/polym16212961

**Published:** 2024-10-22

**Authors:** Diego Mauricio Sánchez-Osorno, Sandra L. Amaya-Bustos, Carlos Molina-Ramírez, María Camila López-Jaramillo, Julián Paul Martínez-Galán

**Affiliations:** 1 Grupo de Investigación e Innovación Ambiental (GIIAM), Institución Universitaria Pascual Bravo, Cl. 73, No 73a-226, Medellín 050034, Colombia; diego.sanchez@pascualbravo.edu.co (D.M.S.-O.); m.lopezja@pascualbravo.edu.co (M.C.L.-J.); 2 Grupo de Investigación Alimentación y Nutrición Humana (GIANH), Escuela de Nutrición y Dietética, Universidad de Antioquia, Cl. 67, No 53-108, Medellín 050010, Colombia; 3 Grupo de Investigación e Innovación en Energía (GIIEN), Institución Universitaria Pascual Bravo, Cl. 73, No 73a-226, Medellín 050034, Colombia; sandra.amaya@pascualbravo.edu.co; 4 Grupo de Investigación en Química y Bioprospección de Productos Naturales (QUIBIP), Universidad del Magdalena, Cl. 29H3 No 22-01, Santa Marta 470004, Colombia; cmolinar@unimagdalena.edu.co

**Keywords:** bacterial nanocellulose, vitamin B complex, thermal protection, isothermal absorption, spray drying, encapsulation

## Abstract

Bacterial nanocellulose has been commonly used as a gelling or stabilizing agent in the food industry and as an excipient in pharmacology. However, due to its physical and chemical properties, such as its high degradation temperature and the ease with which it can interact with other molecules, bacterial nanocellulose has been established as a material with great potential for the protection of bioactive compounds. This research shows the capacity of bacterial nanocellulose to establish interactions with B vitamins (B1, B2, B3 and B12) through different sorption isotherms, mainly by means of the BET, GAB and TSS models. First, the degradation of the vitamin B complex, which mostly occurs upon heating, is minimized in the presence of BNC, herein proposed as a thermal stabilizer. Secondly, BNC is shown to bind to micronutrients and act as dietary fiber. BNC acts as a thickening and water-binding agent. The effects of BNC are determined to occur as an encapsulation system that facilitates affinity adsorption in mono- and multilayers. Finally, bacterial nanocellulose was used as an encapsulating agent for the vitamin B complex by spray drying. It is demonstrated that BNC is a very successful new nanomaterial for encapsulation, with a high level of adsorption, and for the protection of hydro-soluble vitamins. BNC has shown great potential to adsorb vitamins B1, B2, B3 and B12 owing to their hydroxyl groups, which are responsible for its water or vitamin sorption. Due to the features of bacterial nanocellulose, it is possible to use it as a raw material in the food industry to protect micronutrients during the thermal process.

## 1. Introduction

Food fortified with highly bioavailable micronutrients has become common in order to address major deficiencies in the human diet affecting development. Among the major micronutrients, vitamins are important substances, most of which are not synthesized by our body but are closely involved in biochemical functions in living organisms. Thus, they have to be supplied through diet. An insufficient supply of vitamins can lead to diseases such as scurvy, pellagra, ariboflavinosis, dermatitis and enteritis [1].

Many vitamins added in foodstuff may be completely or partially denatured or damaged during cooking or processing. For instance, compared to retinol, thiamine, riboflavin and niacin, Vitamin C, folate and vitamin B6 are less stable under high-temperature processing. A loss of vitamins also occurs during chilling, heating, reheating and storage [2]. While micronutrient losses are mainly due to excess heat and moisture changes, operations inherent to processing such as trimming, cutting, chopping, slicing, washing and soaking are also important factors [3]. Thus, precautionary measures are required in order to preserve vitamin concentration and functions during food processing and storage. A common way to offset such challenges is to incorporate an excess amount of vitamins, which increases costs. Obviously, better approaches are required, and among these, encapsulation is an alternative that has been effective for food fortification at different scales [4].

Encapsulation enables the protective ability of biomolecules, including vitamins, and facilitates their controlled release through the gastrointestinal tract. Encapsulation can protect nutrients from the following: (1) temperature and pH changes; (2) microbial degradation; (3) degradation by undesirable interactions with air and light; and (4) contamination [5]. In addition, encapsulation prevents unwanted reactions between vitamins and other food components, which otherwise would decrease their bioavailability. In sum, encapsulation offers a mechanism to create a barrier to separate the biocomponent (usually as a core in liquid, solid or gas forms) from the matrix components (usually as a shell or coating) and also allows for the design of controlled release profiles [5].

Varying based on their use, coating materials and encapsulation techniques have been developed, yielding a set of physical–chemical characteristics (particle size, porosity, density, flowability, integrity, reactivity, stability, etc.) and release properties. A wide variety of coating or encapsulating materials exist, but those most commonly used in the food industry include maltodextrin, carboxymethyl cellulose (CMC) and guar gum [6,7]. A wide range of encapsulation techniques are available, which can be classified based on the mechanisms for the formation of particles, including physical processes (such as spray drying, spray chilling/cooling, extrusion and fluidized bed coating) and chemical processes (such as coacervation, gelation, co-crystallization, molecular inclusion and interfacial polymerization). In general, the physical techniques are less expensive and easy to scale, with spray drying being one of the most frequently used in the industry. In this latter case, a solvent is evaporated from a liquid suspension, forming solid particles between five and one-hundred fifty µm that are covered with water-soluble polymers [4,8,9].

Vitamins commonly used in encapsulated forms via spray drying include vitamins E [10], C [11], D [12], riboflavin [13], thiamine and pyridoxine [14], nicotinic acid [15], cyanocobalamin [16] and folic acid [17]. Their protection has been targeted in relation to moisture, photodegradation, diffusion patterns, stability, transport and oral delivery. Thermal protection, however, has not received the same level of attention.

Nanotechnology has the potential to improve processed food’s taste, aroma and functional properties and can facilitate healthier and improved packaging [18]. It is also instrumental in the design of drug release control systems. In this context, different types of nanocelluloses have been proposed for applications in the food industry [19] and their safety in this area has been proven by different toxicity test [20] and in controlled drug release systems [21,22]. Among these systems, bacterial nanocellulose (BNC) has the same basic and morphological characteristics of vegetal nanocellulose and represents a food-safe polymeric material produced biosynthetically by bacteria belonging to the *Komagataeibacter* genus. BNC is considered a chemically pure nanomaterial comprising nanoribbons (40–60 nm in width) produced aerobically as a pellicle on top of a culture medium [23]. In this form, BNC displays a hydrogel behavior with a rheology that is suitable for use in food industries and commonly found in dessert delicacies such as nata de coco; as a multifunctional food and beverage ingredient; and as a thickener, stabilizer and texture modifier [24,25]. BNC’s nanostructure, thermal stability and mechanical properties in encapsulation systems have not been explored. As such, this work addresses the design and development of an encapsulation system based on the spray drying of bacterial nanocellulose for heat protection and vitamin fortification. Layered assemblies are unveiled by sorption isotherms that indicate the involved affinity and interactions between BNC and the B vitamin complex. Overall, a viable solution to nutritional challenges in disadvantaged communities in Latin America is demonstrated using local bioresources.

## 2. Materials and Methods

**Figure 1:**
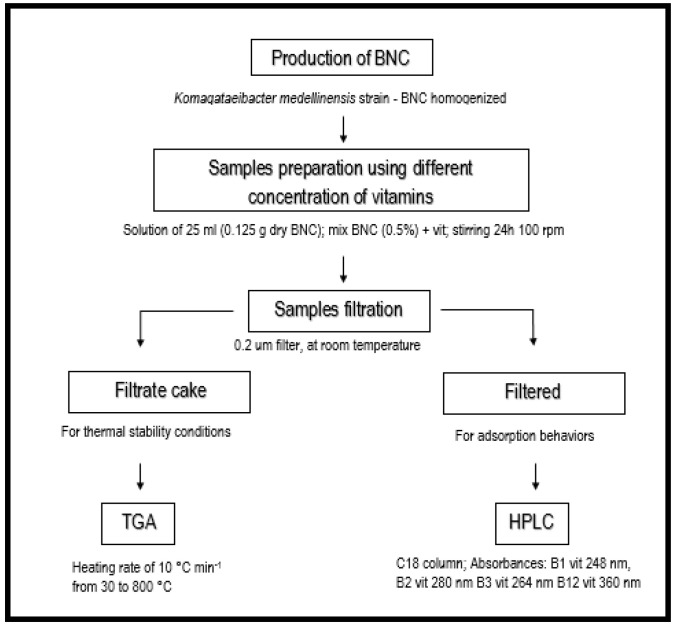
Process diagram: Investigative activities for the determination of thermal protection ability of BNC in vitamins.

Here, bacterial nanocellulose (BNC) was produced by fermentation using *Komagataeibacter medellinensis* strain. The culture media for fermentation were prepared using agroindustrial (fruit) residues. The mix of fruits was homogenized (one part of fruit per 2 parts of water) in a SKYMSEM LAR-04MB blender (Skymsem, Brusque, Brazil), filtered through nylon strainers with different open areas, adjusted to a pH of 3.5 with acetic acid and sterilized at 121 °C for 20 min. Fermentation was carried out by adding 10 vol% inoculum to the different media and statically incubating the system at 28 °C for 60 days. The BNC pellicles collected from the surface were washed with water, treated for 14 h in a 5 wt% KOH solution and rinsed until reaching neutral pH. The pellicles were homogenized in water to extract the nanoribbons using a Masuko MKCA6-2 grinder (Masuko Sangyo, Kawaguchi, Japan) with MK GC 6–120 SD disc. Four different disc gap sizes were used with the nanocellulose passing though each gap four times. Before its use, BNC was sterilized under the same conditions as those indicated above. The block diagram in Figure 1 summarizes the experimental approach used in this work to determine the vitamin adsorption behaviors of BNC and the thermal protection conditions provided by the BNC to each vitamin.

### 2.1. Isothermal Adsorption of Vitamin B in BNC Solution

To determine the thermodynamic affinity of each of the vitamins for the surface of the BNC and to describe their adsorption behavior, isothermal adsorption studies were conducted. Aqueous BNC dispersion (25 mL at 0.5 wt% *w*/*v* dry BNC) was mixed with vitamin B complex, which was loaded at different levels, ranging between the maximum water solubility to 1/10 of this value. The vitamin amount on a dry BNC basis for each mixture is shown in Table 1.

The amount of vitamin adsorbed in BNC was calculated according to the difference between the initial and final concentration in solution using Equation (1):

(1) $$q_{C_{w}}=[\left(C_{o}-C_{w}\right)*\frac{V}{M}]$$

where $q_{C_{w}}$ is the amount of vitamin adsorbed in bacterial nanocellulose (mg/g); C_o_ is the concentration of vitamins in the initial solution (mg/L); C_w_ is the final concentration of vitamins in solution (mg/L); V is the volume of the adsorption medium (L); and M is the (dry) mass of BNC (g). $q_{C_{w}}$ and C_w_ values for each vitamin were plotted and fitted according to different empirical isotherms. The best fit was used to calculate *q_max_*, *C*, *K* and *h,* which corresponded to a monolayer maximum capacity (mg/g), the adsorption monolayer thermodynamic constant, adsorption multilayer thermodynamic constant and number of layers in the second sorption stage, respectively, using equations from Table 2. A nonlinear solver method in Matlab 7.12.0. (R 2011a) was used for this purpose.

The change in Gibb’s free energy thermodynamic parameter ΔG° was calculated using C obtained from BET in GAB and TSS equations (Table 2) and Equation (2):

(2) ∆G°=−RTlnC

where R is the gas constant, T is the absolute temperature (K) and C is an equilibrium monolayer adsorption thermodynamic constant.

### 2.2. High-Performance Liquid Chromatography (HPLC)

For HPLC analysis, a 250 × 4.6 mm C18 column (Waters Milford, MA, USA) was used with a mobile phase consisting of 50:47.5:2.5 solution of methanol, acetonitrile and water flowing at 1.0 mL/min. Vitamin absorbance was determined at the following wavelengths: B1, 248 nm; B2, 280 nm; B3, 264 nm; and B12, 360 nm. The injection volume was 50 μL. Samples were diluted to fit to the specific calibration curve using B1 and B12, which were diluted in 0.1 N HCl. B2 and B3 were diluted in 0.02 N acetic acid.

### 2.3. Thermogravimetric Analysis (TGA)

To determine the thermal protection conferred by the BNC to each vitamin, different dried filtrate cakes were used for thermogravimetric analyses (10 mg each) using a Mettler Toledo TGA/SDTA 851e instrument (Mettler Toledo, Columbus, Ohio, USA) under nitrogen flow of 40 mL min^−1^ and 10 °C min^−1^ heating rate. The samples were heated from 30 to 800 °C. The thermal decomposition temperature was taken as the onset of significant (≥ 0.5%) weight loss.

### 2.4. Vitamin Encapsulation in BNC

The vitamins were encapsulated in BNC by using a Buchi B-290 mini spray dryer (Büchi Labortechnik AG, Flawil, Switzerland) with a standard 0.5 mm nozzle under the following conditions (selected considering preliminary studies): dispersion and air flow rates, air pressure, inlet temperature and outlet temperature were set to 5 mL/min (25%), 35 m^3^/h (90%), 6.0 bar, 200 °C and 100 °C, respectively. Vitamin encapsulated samples were collected, sealed and stored in dry conditions at room temperature until morphology analysis.

### 2.5. Attenuated Total Reflection Fourier-Transform Infrared Spectroscopy (ATR-FT-IR)

Encapsulated vitamins were analyzed by FT-IR spectra on a Nicolet 6700 spectrophotometer (Thermo Electron Corporation, Waltham, MA, USA) in the 4000–400 cm^−1^ range ATR with a diamond crystal. The spectra were recorded with a resolution of 4 cm^−1^ and an accumulation of 64 scans.

### 2.6. Scanning Electron Microscopy (SEM)

SEM was used to observe the morphology and particle size distribution of BNC-encapsulated vitamins using a JEOL JSM 6490 LV (Jeol Ltd., Tokyo, Japan) operating at 20 kV. Before observation, the samples were coated with gold/palladium using an ion sputter coater. The size distribution was determined using Image J software version 1.54. Thirty particles were selected from each image and approximated to ellipses. The largest and shortest diameters were measured and averaged.

### 2.7. Transmission Electron Microscopy (TEM)

Samples were dispersed in distilled water and sonicated. Drops of each suspension were deposited onto glow-discharged carbon-coated electron microscopy grids and negatively stained with 2 wt% uranyl acetate aqueous solution for ten minutes in the absence of light. All samples were observed using an FEI Tecnai G2 F20 (FEI Company, Hillsboro, OR, USA) microscope operating at an acceleration voltage of 200 kV.

## 3. Results and Discussion

### 3.1. Sorption Isotherms

**Figure 2:**
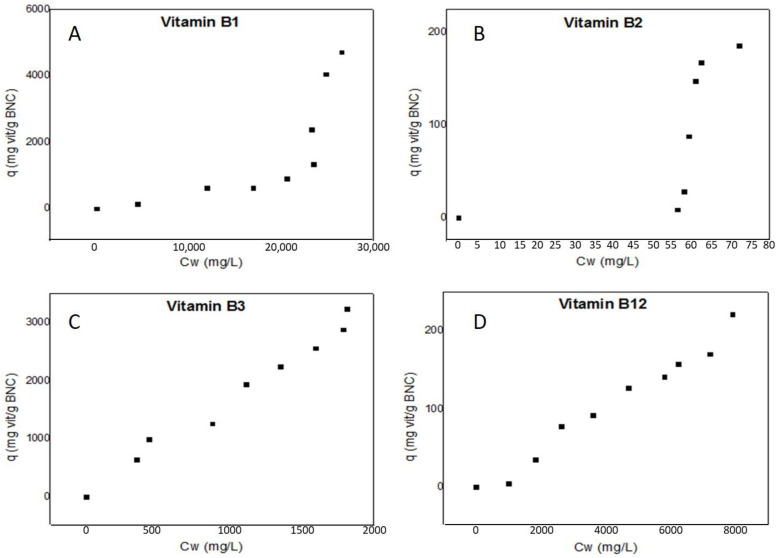
Experimental isotherms for vitamin sorption in BNC at 298 K and neutral pH: (**A**)—Thiamine hydrochloride. (**B**)—Riboflavin. (**C**)—Niacin. (**D**)—Cyanocobalamin.

The adsorption data for each vitamin B (B1, B2, B3 and B12) in BNC are shown in Figure 2 in order to determine their affinity. The shape of the adsorption isotherms was used to categorize the adsorption types according to the IUPAC isotherm classification system as types V and IV. All samples except for B3 indicated a type V isotherm; B3 was type IV. The profiles are consistent with isotherms associated with hydrophilic and porous polymers such as nanocellulose [26,27,28,29,30]. The BET, GAB and TSS isothermal models, which are commonly used to empirically describe multilayer sorption, were used to fit the experimental data. The TSS model was chosen for further analysis, given its higher correlation coefficient (R^2^).

The TSS model uses the following constants: q_max_ (the monolayer maximum capacity), C (the adsorption monolayer thermodynamic constant), K (the adsorption multilayer thermodynamic constant) and h (the number of layers in the second sorption stage). The values determined for each vitamin are shown in Table 3. The K values obtained in this experiment are in agreement with Timmerman [31,32], in which K < one. The low values for the adsorption multilayer thermodynamic constant (K) for all the vitamins are evidence of the weak bond energy between vitamins and finite multilayer formation [26,31]. In accordance with Timmerman, the K values below one indicate the existence of less than 10 sorption layers at saturation; similarly, the obtained low values for the h constant were in accordance with the K values, thus showing that lower values of parameter h are related with a smaller second sorption stage [32].

The free Gibbs energy (ΔG°) was obtained by using the C constant in Equation [33]. The high negative values for the free Gibbs energy showed a spontaneous adsorption process for all the vitamins. These values are shown in Table 3. As the experiment was conducted at low temperatures and the reaction was spontaneous, it is possible to deduct that ΔH < 0 and ΔS < 0, showing an exothermic reaction and suggesting a more orderly reorganization, respectively [34]. Given that the free Gibbs energy was negative and the experiment was conducted at low temperatures, it is possible to conclude that ΔH > T∆S. Therefore, the B vitamin complex has an affinity for the BNC’s surface and the adsorption process is spontaneous.

In Figure 2, the first inflection point observed in the zone of low concentration (C_w_) in the range of 10 to 70 mg/L was used to determine the saturation of the first layer. The monolayer condition was calculated for all vitamins by the q_max_ from the TSS model and is around 55 mg vit B1/g BNC. The h value for this vitamin was 0.7. This value is equivalent to 70% of the total amount of the vitamin required to form the first layer [35]. For B2, the monolayer (q_max_) is formed with 19 mg vit B2/g BNC. The value of h = 5 suggests the possibility to form five multilayers. Regarding the B3 adsorption isotherm analysis, a monolayer (q_max_) is formed around 60 mg vit B3/g BNC, followed by a multilayer expressed as h, exhibiting two complete layers and an incomplete third layer. Regarding the B12 adsorption isotherm analysis, the h value of 4.11 shows that multilayers were formed, which is evidence of the interaction between cellulose and the vitamin as well as between vitamins. In this vitamin, a monolayer is formed with 20 mg vit B12/g BNC. The adsorption between the vitamin and bacterial nanocellulose to form the first layer is different for each vitamin, possibly due to the molecule sizes used. The following results were obtained: B1: 265.356 g/mol; B2: 376.36 g/mol; B3: 123.1094 g/mol; and B12: 1355.365177 g/mol. Moreover, each molecule has different quantities of hydroxyl or nitrogen groups interacting with the OH groups of bacterial cellulose by hydrogen bonds.

### 3.2. Thermograms

**Figure 3:**
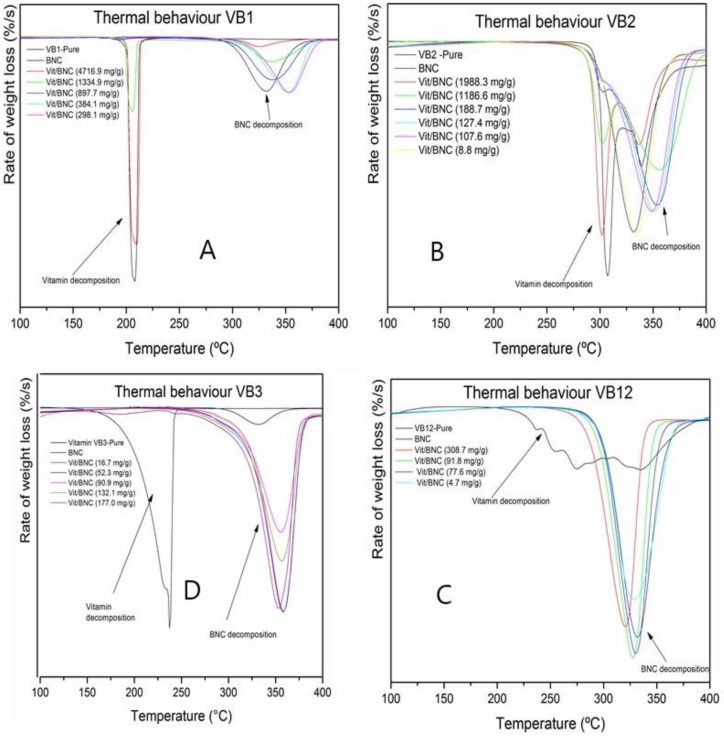
DTG of different concentrations of vitamins and bacterial nanocellulose: (**A**): Vitamin B1-BNC; (**B**): Vitamin B2-BNC; (**C**): Vitamin B3-BNC; (**D**): Vitamin B12-BNC.

The thermal protection conferred by the BNC to each vitamin was evaluated, and the DTG results of the vitamins, BNC and different vitamin:BNC ratios are presented in Figure 3. The pure vitamins reveal a maximum degradation temperature with values between 200 and 275 °C (Table 4), while the maximum temperature of the degradation of the cellulose is 350 °C. On the other hand, the DTG thermograms show two degradation areas at high vitamin:BNC ratios: the first one due to the degradation of the vitamin, which does not gain protection from the BNC, and the second one indicating the degradation of the BNC (Figure 3). However, as the vitamin:BNC ratio decreases, the first degradation zone begins to disappear until it is possible to find a ratio in which both components are degraded in the same zone (close to the maximum degradation temperature of the cellulose). This condition is shown for each vitamin in Table 4. Under these conditions, it is possible to conclude that the cellulose imparts a greater thermal stability to the different vitamins by moving their maximum degradation temperatures to higher temperature values, and this BNC characteristic can be harnessed for the preservation of the stability of vitamins during different stages of food processing, preventing the loss of nutritional quality.

The BNC was shown to protect the vitamins at the following concentrations: 61 mg of vitamin B1/g BNC; 107 mg of vitamin B2/g BNC; 132 mg of vitamin B3/g BNC; and 91 mg of vitamin B12/g BNC. These concentrations of each vitamin in BNC coincide with the vitamin amount found in the monolayer. Some layers are expressed by the h values found via the TSS model (see Table 3) and are discussed above. We speculate that the above amounts indicate that BNC can protect vitamins that are directly bound to its surface in monolayers, with some vitamins deposited in adjacent layers. For vitamin B1, thermal protection was conferred with 61 mg and a monolayer was formed with 55 mg. For vitamin B2, thermal protection was conferred with 107 mg and a monolayer was formed with 19 mg, followed by five multilayers. For vitamin B3, the maximum thermal protection was conferred with 132 mg, and a monolayer was formed with 60 mg, followed by two more layers. Finally, for vitamin B12, thermal protection was conferred with 91 mg and a monolayer was formed with 20 mg, followed by four multilayers (Table 4).

### 3.3. Chemical Analyses

**Figure 4:**
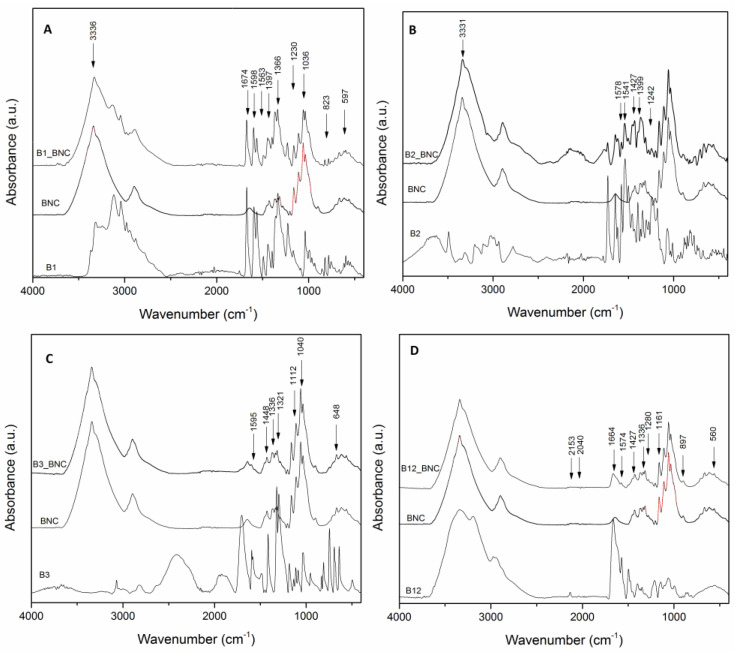
FTIR difference spectra for region between 4000 and 400 cm^−1^; (**A**): Bacterial nanocellulose (BNC) and thiamin (B1) mixed with BNC; (**B**): Bacterial nanocellulose (BNC) and riboflavin (B2) mixed with BNC; (**C**): Bacterial nanocellulose (BNC) and nicotinic acid (B3) mixed with BNC; (**D**): Bacterial nanocellulose (BNC) and cianocobalamin (B12) mixed with BNC.

The BNC, vitamins and BNC–vitamin systems were subjected to attenuated total reflection Fourier-transform infrared spectroscopy (Figure 4). It was possible to note the presence of the individual compounds from their typical bands (BNC, B1, B2, B3 and B12). For the pure BNC, typical bands of the IR spectra were registered [36,37,38]. Bands in the range of 3800–3000 cm^−1^ are related to the sum of the valence vibrations of H-bonded OH and intramolecular and intermolecular hydrogen bond [39]. The peaks at 2850 cm^−1^ and 2918 cm^−1^ come from CH_2_ and CH symmetric and asymmetric stretching, respectively [39]. Moreover, the spectra showed the characteristic bands of nanocellulose with strong bands at 1429 and 1111 cm^−1^ assigned to CH_2_ symmetrical bending and C–O bond stretching, respectively. Other interesting bands for this research paper are as follows: 1375 cm^−1^ (CH bending); 1335 cm^−1^ (O–H in-plane bending); 1315 cm^−1^ (CH_2_ wagging); 1277 cm^−1^ (CH bending); and 1225 cm^−1^ (O–H in-plane bending) [40,41].

Interactions between the BNC and vitamins (B1, B2, B3 and B12) could be possible due to the hydroxyl groups present in the BNC, which are responsible for both the water’s and vitamins’ sorption. Nonetheless, strong hydrogen bonds exist in the nanocellulose, and for this reason, only 40–60% of the hydroxyl groups of the bacterial nanocellulose are associated with the vitamins [42].

By comparing the spectra of B1 and BNC (Figure 4A), a wide absorption range in the region of 3800 cm^−1^~3000 cm^−1^ was noted. This peak is typical for free O-H stretching bands [43]. In addition, the shift of the bands between 550 cm^−1^ and 1700 cm^−1^ is the result of the interaction between carbohydrates of nanocellulose and thiamine [41]. The amine and OH groups of B1 interact with the O-H groups of bacterial nanocellulose, forming hydrogen bonds, which are considered secondary interactions [44]. These interactions are evident due to shifts in the bands involved in hydrogen bonding: CH deformation at 1405 cm^−1^ (CH_2_-CH_2_)+OH; pyrimidine ring stretching at 1656 cm^−1^, 1592 cm^−1^, 1542 cm^−1^ and 1381 cm^−1^; out-of-plane pyrimidine ring and thiazole ring bending at 820 cm^−1^; and out-of-plane NH deformation (NH_2_) at 582 cm^−1^ and 573 cm^−1^ [39,45].

Riboflavin (vitamin B2) is a hydrophilic compound due to its four –OH groups; however, its noted reduced affinity with water could explain the water retention behavior of the crosslinked nanocellulose construct observed in the dry sample [46]. In Figure 4B, a broad band of high intensity was observed in the region centered at 3336 cm^−1^ of the B2-BNC spectra, exhibiting the presence of OH groups [47]. The FTIR spectra of B2-BNC, with four bands at 1397 cm^−1^, 1545 cm^−1^, 1579 cm^−1^ and 1646 cm^−1^, were due to the incorporation of riboflavin into the bacterial nanocellulose. The band at 1397 cm^−1^ is characteristic of C=N [48]. Martins 2014 [49] indicated that bands at 1545 cm^−1^, 1579 cm^−1^ and 1583 cm^−1^ could be attributed to stretching vibrations of C=N and C=N bonds in the isoalloxazin unit rings. These bands are shifted, as can be seen by comparing the spectra. Meanwhile, a shift in the band at 1548 cm^−1^ was due to the stretching vibrations of the C=N bonds for the isoalloxazin ring of the carbonyl group (C_23_=O) [49]. It is possible to note a shift at 1425 corresponding to the deformation vibration of the hydroxyl group [49]. Another shift is noted at 1242, corresponding to the hydrogen bonding interactions of the isoalloxazine system [50].

Figure 4C shows the B3 spectra the characteristic bands of niacin in different regions.

Region I 3500−3000 cm^−1^: 3447 cm^−1^ m: stretch (O_9_–H); 3104 cm^−1^ w: stretch (C_2_–H)—stretch (C_5_–H); 3084 cm^−1^ w: stretch (C_6_–H)—stretch (C_5_–H); 3071 cm^−1^ m: stretch (C_2_–H)—stretch (C_4_–H) + stretch (C_5_–H) + stretch (C_6_–H); and 3042 cm^−1^ w: stretch (C_4_–H) + stretch (C_5_–H) [51].

Region II 1800−1500 cm^−1^: 1708 cm^−1^ s: stretch (C=O); 1595 cm^−1^ s: stretch (N–C_2_) + stretch (C_2_–C_3_)—stretch (C_3_–C_4_)—stretch (C_5_–C_6_); and 1583 cm^−1^ s: stretch (N–C_2_)—stretch (C_2_–C_3_) + stretch (C_3_–C_4_)—stretch (C_5_–C_6_)+ stretch (N–C_6_). The nicotinamide (niacin) spectrum is intense and the functional amide is characteristic, with two bands between 1610 cm^−1^ and 1600 cm^−1^ [51,52].

Region III 1500−1300 cm^−1^: 1488 cm^−1^ m: stretch (N–C_2_) + stretch (C_2_–C_3_)—stretch (C_3_–C_4_)—stretch (C_5_–C_6_); 1449 cm^−1^ w: stretch (N–C_2_)—stretch (C_2_–C_3_)—stretch (C_3_–C_4_) + stretch (C_4_–C_5_)—stretch (C_5_–C_6_)—stretch (NC_6_) + stretch (C_3_–C_7_); 1370 cm^−1^ vw: in-plane deformation vibration (OH) [53]; and 1301 cm^−1^ in-plane deformation (CH) [51,54].

Region IV 1300−500 cm^−1^**_:_** 1298 cm^−1^ in-plane deformation OH + in-plane deformation CH + stretching (C-O); 1112 cm^−1^ in-plane deformation CH + stretching (C-C) + stretching (C-N); 1030 cm^−1^ scissoring ring + stretching ring; 809 cm^−1^ scissoring ring + stretching (C- ring substituent) + scissoring (COO); and 647 cm^−1^ scissoring (COO)+ scissoring ring + in-plane deformation (O-H) [54].

By comparing the spectra of B3 and BNC (Figure 4C), it is possible to note that most of the previous bands observed in the B3 spectra show small wavenumber shifts, especially in the regions of I and II in accordance with small shifts in the band characteristics of the BNC in the same regions. Additionally, the characteristic bands of the BNC spectra in the region of 1700 cm^−1^–500 cm^−1^ are present in the B3-BNC spectra and have the same wavenumber; in contrast, the characteristic bands of the B3 spectra in the same region undergo small wavenumber shifts. These wavenumber shifts and the broadening of the bands for the B3-BNC spectra are taken as evidence of the chemical interactions between vitamin B3 and bacterial nanocellulose and are indicative of miscibility [55]. These shifts could be due to hydrogen bonding in the mix.

For the FTIR spectra of B12-BNC (Figure 4D), the weak bands observed at 2900 cm^−1^, 2120 cm^−1^ and 1890 cm^−1^ correspond to CH, C≡C and C=O, respectively. The characteristic absorption bands of B12 in the range of 900 cm^−1^–1800 cm^−1^ belong to C=O stretching vibration at 1700 cm^−1^; C=C stretching vibration at 1580 cm^−1^ and 1500 cm^−1^; CH bending vibration at 1390 cm^−1^; and C–O stretching vibration at 1220 cm^−1^, 1140 cm^−1^ and 1020 cm^−1^ [56]. As can be seen, the main structure of B12 is maintained by loading on the BNC. The low-intensity peaks of pristine B12, such as the peaks at 2900 cm^−1^, 2120 cm^−1^, 1890 cm^−1^, 1580 cm^−1^ and 1390 cm^−1^, disappeared in the B12–BNC spectrum.

By comparing the B12 and BNC spectra, it is possible to note that both show changes after mixing but these changes are more prominent for the B12 spectra. The main change in the bacterial nanocellulose after mixing is noted in the shift in the wavenumber of the bands: 2160 cm^−1^, 2050 cm^−1^, 1995 cm^−1^ and 1906 cm^−1^. The main change for B12 after mixing is observed as a shift in the wavenumber of the bands between 2200 and 1500 cm^−1^. In this region, characteristic bands of the B12 spectra are present in B12-BNC (Figure 4D). Moreover, it is observed that the original bands of the B12 spectra in the range of 1300 cm^−1^–800 cm^−1^ are weak or very weak in the B12-BNC spectra. These signals refer to 647 cm^−1^ coring ring, 555 cm^−1^ C=N–O, 486 cm^−1^ Co–CN stretching and 460 cm^−1^ Co–N stretching.

Because of the low intensity of the FTIR, the B12 loading on the BNC surface does not cause a remarkable change in the B12-BNC spectra; therefore, the probable network structure is not detectable by FTIR, as reported by Gharagozlou.

It is possible that interactions between bacterial nanocellulose and vitamins occur, particularly between the OH group with six carbon atoms in the bacterial nanocellulose and the OH groups of each vitamin due to being more exposed than the OH groups with two, three and four carbon atoms. In addition, these interactions may particularly occur between the OH and N groups (amine and amide) of each vitamin and the OH groups of the bacterial nanocellulose. All the mixes that show shifts in wavenumbers correspond to groups that involve OH and/or N groups.

### 3.4. Imaging of Spray-Dried BNC-Encapsulated Vitamins

**Figure 5:**
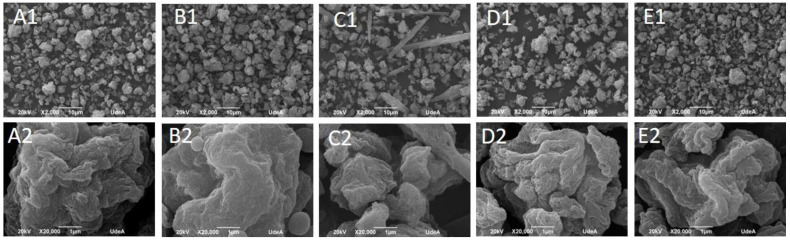
SEM micrographs of bacterial nanocellulose and bacterial nanocellulose with different microencapsulated vitamins. Up (X2000) and down (X20,000). (**A1**,**A2**): BNC; (**B1**,**B2**): vitamin B1 (thiamine); (**C1**,**C2**): vitamin B2 (riboflavin); (**D1**,**D2**): vitamin B3 (niacin); (**E1**,**E2**): vitamin B12 (cyanocobalamin).

The relation between the BNC and vitamins was found by a TGA in which filtrated cakes were used to produce microcapsules by spray drying. The SEM images shown in Figure 5 indicate a spheroidal morphology with sizes between ~two and ~eight µm (with the most frequent particle size being between 4 and 5 µm for all the vitamins; Table 5). No aggregates with sizes greater than 10 microns were observed.

By comparing the different micrographs at X2000, it is possible to note bacterial fibrils that are subdued in the BNC–vitamin combinations, indicating that the surface of the bacterial nanocellulose is modified by the vitamins. This observation is evidence of the sorption of vitamins in bacterial nanocellulose as well as monolayer and multilayer formation as shown by the sorption isotherm. Interactions between the bacterial nanocellulose and the different vitamins were observed by FTIR. For vitamin B2, it is possible to note how an excess of this vitamin leads to the formation of needles that are not protected (as confirmed by the TGA).

**Figure 6:**
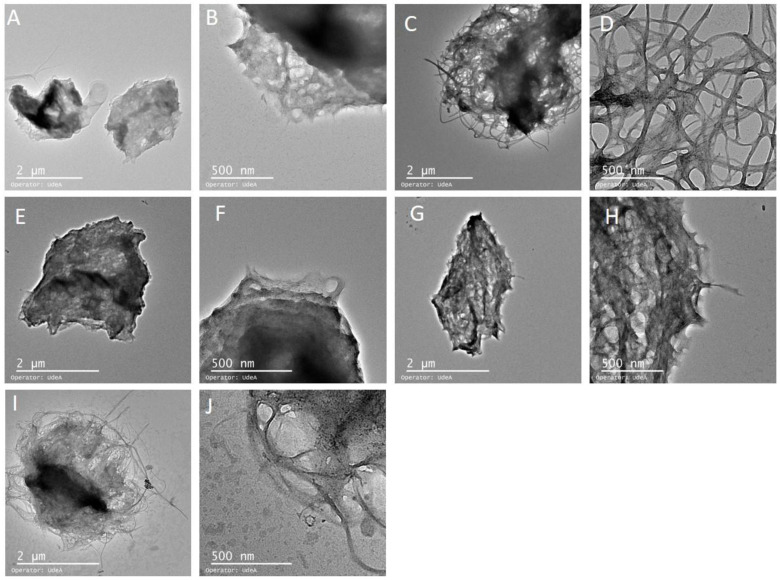
Micrographs at 2 µm and 500 nm of bacterial nanocellulose (**A**,**B**), vitamin B1 (**C**,**D**), vitamin B2 (**E**,**F**), vitamin B3 (**G**,**H**) and vitamin B12 (**I**,**J**).

TEM images recorded from negatively stained samples are shown in Figure 6, which includes a particle appearance that suggests the encapsulated vitamins are in a web formed by nanoribbons of the bacterial nanocellulose. The nanoscale of the bacterial cellulose increases the superficial area, allowing for a greater degree of interaction with the vitamins. The web is quite different for each vitamin, possibly due to the specific chemical and physical characteristics of each vitamin. This web of bacterial nanocellulose could be responsible for the improved thermal behavior of the vitamins in the web, as confirmed by the TGA and the interactions that were observed by FTIR and the sorption isotherms.

## 4. Conclusions

In the present investigation, it is demonstrated that BNC is a nanomaterial that could be used alone as an encapsulating agent. BNC showed high levels of adsorption and provided thermal protection to water-soluble vitamins such as the vitamin B complex. In this investigation, BNC showed great potential to adsorb vitamins B1, B2, B3 and B12 due to its hydroxyl groups which are responsible for its water or vitamin sorption, as confirmed by FTIR and adsorption modelling (61 mg of vitamin B1/g BNC; 107 mg of vitamin B2/g BNC; 132 mg of vitamin B3/g BNC; and 91 mg of vitamin B12/g BNC). Furthermore, the negative free Gibbs energy with ΔH > T∆S confirms the interactions between the BNC and the vitamins and the interactions between the vitamins due to spontaneous and exothermic reactions. Finally, the TGA results confirm that BNC can thermally protect the vitamin B complex in both monolayers and multilayers. BNC improved the maximum temperature degradation for each vitamin (B1: 207 °C to 340 °C; B2: 309 °C to 334 °C; B3: 249 °C to 360 °C; and B12: 275 °C to 329 °C).

Due to the characteristics of bacterial nanocellulose, it is possible to use it as an encapsulating agent in the food industry, with the aim of thermally protecting bioactive compounds, which can be degraded in the industrial processing stages in which the use of high temperatures is mandatory (i.e., pasteurization or evaporation), in addition to its nutritional contribution as dietary fiber.

## Figures and Tables

**Table 1:** Percentage of vitamin B complex components in BNC in mixtures used for isothermal adsorption studies.

Vitamin	Vitamin/BNC Dry Basis (wt%)									
B1	40	36	32	30	28	24	20	16	12	8
B2	0.056	0.050	0.045	0.042	0.039	0.034	0.028	0.022	0.017	0.011
B3	14.4	12.96	11.52	10.8	10.08	8.64	7.2	5.76	4.32	2.88
B12	10.4	9.36	8.32	7.8	7.28	6.24	5.2	4.16	3.12	2.08

**Table 2:** Isotherm models used to determine the thermodynamic affinity of each of the vitamins for the surface of the BNC, where q_max_: monolayer maximum capacity (mg vit/g BNC); C_w_: equilibrium concentration (mg/L); C: adsorption monolayer thermodynamic constant; K: adsorption multilayer thermodynamic constant; and h: number of layers in the second sorption stage.

Model	Equation
BET (Brunauer–Emmett–Teller) Constants: $q_{max}\;\mathrm{and}\;C$	$q_{\left(C_{w}\right)}=\frac{q_{max}*C*C_{w}}{\left[\left(1-C_{w}\right)*\left(1+\left(C-1\right)*C_{w}\right)\right]}$ (3)
GAB (Guggenheimer–Anderson–de Boer) Constants: $q_{max};C\;\mathrm{and}\;K$	$q_{\left(C_{w}\right)}=\frac{q_{max}*C*K*C_{w}}{\left[\left(1-K*C_{w}\right)*\left(1+\left(C-1\right)*{K*C}_{w}\right)\right]}$ (4)
TSS (Three Sorption Stages) Constants: $q_{max};C\;;\;K\;\mathrm{and}\;h$	$q_{\left(C_{w}\right)}=\frac{q_{max}*C*K*C_{w}*H*H\prime }{\left[\left(1-{K*C}_{w}\right)*\left(1+\left(C*H-1\right)*{K*C}_{w}\right)\right]}$ (5) where $H=1+\left[\frac{1-K}{K}\right]*\frac{\left[{\left(K*C_{w}\right)}^{h}\right]}{1-C_{w}}$ $H^{\prime }=1+\left[\frac{1-K}{K}\right]*\left[\frac{1-K*C_{w}}{1-C_{w}}\right]*[h+\left(1-h\right)*C_{w}]$

**Table 3:** Parameters of TSS isotherm models for each vitamin, where V_mG_: monolayer maximum capacity (mg vit/g BNC); C_g_: adsorption monolayer thermodynamic constant; R^2^: correlation coefficient; K: adsorption multilayer thermodynamic constant; h: number of layers in the second sorption stage; ΔG: free Gibbs energy.

Model	Parameter	B1	B2	B3	B12
BET	V_mB (mg vit/g BNC)_	−40	43	70	65
	C_B_	1	269	228	20.5
	R^2^	0.9000	0.4572	0.9272	0.9187
	ΔG	0.0000	−13,861.8671	−13,452.1448	−7483.6262
GAB	V_mG (mg vit/g BNC)_	55	53	79	60
	C_g_	213	219.49	138	35.5
	K	3.49 × 10^−5^	0.008	4.9 × 10^−4^	3.9 × 10^−5^
	R^2^	0.9418	0.5100	0.9774	0.9633
	ΔG	−13,283.5306	−13,357.8967	−12,208.1250	−8844.1359
TSS	V_mG (mg vit/g BNC)_	55	43	81	60
	C_g_	213	219.49	157.4	20.5
	K	3.35 × 10^−5^	0.8	4.90 × 10^−4^	3.90 × 10^−5^
	h	3.42 × 10^1^	20	1.328	0.11
	R^2^	0.9416	0.9560	0.9778	0.9811
	ΔG	−13,283.5306	−13,357.8967	−12,534.0298	−7483.6262

**Table 4:** Maximum degradation temperature for vitamins B1, B2, B3 and B12, bacterial nanocellulose and different vitamin:BNC mixtures.

Substance	Maximum Degradation Temperature (°C)	Substance	Vitamin:BNC Ratio (mg vit/g BNC)	Maximum Degradation Temperature (°C)
Bacterial nanocellulose	350	Bacterial nanocellulose/Thiamine	61	340
B1	207			
B2	309	Bacterial nanocellulose/Riboflavine	107	334
B3	249	Bacterial nanocellulose/Niacin	132	360
B12	275	Bacterial nanocellulose/Cyanocobalamin	91	329

**Table 5:** Results of the particle size analysis using Image J software with micrographs at X2000 and X20000 for each vitamin and bacterial nanocellulose.

	Particle Size (µm) Min—Max	Most Frequent Particle Size (µm)
BNC	2.500–7.528	4–5
BNC + B1	2.590–7.273	4
BNC + B2	2.804–6.371	5
BNC + B3	2.289–7.937	4–5
BNC + B12	2.992–7.523	4–5

## Data Availability

The original contributions presented in the study are included in the article, further inquiries can be directed to the corresponding author.
